# Metabolites, Nutrients, and Lifestyle Factors in Relation to Coffee Consumption: An Environment-Wide Association Study

**DOI:** 10.3390/nu12051470

**Published:** 2020-05-19

**Authors:** Mohamed A. Elhadad, Nena Karavasiloglou, Wahyu Wulaningsih, Konstantinos K Tsilidis, Ioanna Tzoulaki, Chirag J Patel, Sabine Rohrmann

**Affiliations:** 1 Institute for Medical Information Processing, Biometry and Epidemiology, Ludwig Maximilian University of Munich, 81377 Munich, Germany; M.Elhadad@live.com; 2 Research Unit of Molecular Epidemiology, Institute of Epidemiology, Helmholtz Zentrum München, German Research Center for Environmental Health, 85764 Neuherberg, Germany; 3 Division of Chronic Disease Epidemiology; Epidemiology, Biostatistics and Prevention Institute (EBPI), University of Zurich, 8091 Zurich, Switzerland; nena.karavasiloglou@uzh.ch; 4 MRC Unit for Lifelong Health and Ageing at University College London, London WC1E 6BT, UK; w.wulaningsih@ucl.ac.uk; 5 Department of Hygiene and Epidemiology, University of Ioannina School of Medicine, 45110 Ioannina, Greece; k.tsilidis@imperial.ac.uk (K.K.T.); itzoulak@uoi.gr (I.T.); 6 Department of Epidemiology and Biostatistics, School of Public Health, Imperial College London, London SW7 2BU, UK; 7 Department of Biomedical Informatics, Harvard Medical School, Boston, MA 02115, USA; Chirag_Patel@hms.harvard.edu

**Keywords:** coffee consumption, environment wide association study, metabolites, nutrients, lifestyle, National Health and Nutrition Examination Survey (NHANES)

## Abstract

Coffee consumption has been inversely associated with various diseases; however, the underlying mechanisms are not entirely clear. We used data of 17,752 Third National Health and Nutrition Examination Survey participants to investigate the association of 245 metabolites, nutrients, and lifestyle factors with coffee consumption. We used data from the first phase (*n* = 8825) to identify factors with a false discovery rate of <5%. We then replicated our results using data from the second phase (*n* = 8927). Regular coffee consumption was positively associated with active and passive smoking, serum lead and urinary cadmium concentrations, dietary intake of potassium and magnesium, and aspirin intake. In contrast, regular coffee consumption was inversely associated with serum folate and red blood cell folate levels, serum vitamin E and C, and beta-cryptoxanthin concentrations, Healthy Eating Index score, and total serum bilirubin. Most of the aforementioned associations were also observed for caffeinated beverage intake. In our assessment of the association between coffee consumption and selected metabolites, nutrients, and lifestyle factors, we observed that regular coffee and caffeinated beverage consumption was strongly associated with smoking, serum lead levels, and poorer dietary habits.

## 1. Introduction

Coffee is the most widely consumed beverage worldwide [1,2], and coffee and caffeinated beverages are some of the most studied beverages nowadays. Since its discovery, drinking coffee has been a growing trend around the globe. Such popularity has cultivated questions on the health effects of its consumption. However, findings in the literature are contradicting. On one hand, coffee has been associated with lower mortality risk [3,4,5,6] regardless of the drinkers’ genetically determined caffeine metabolism capacity [7]. Additionally, coffee has been associated with decreased risk of dementia and Alzheimer’s disease [8,9], Parkinson’s disease [10], type 2 diabetes [11,12], cardiovascular disease (CVD) [13] and certain cancers such as colorectal [6,14], endometrial [6,15], prostate [6,16], and liver cancer [17]. In contrast, studies have reported positive associations between coffee consumption and the risk of some cancers, namely gastric [18,19] and laryngeal cancers [20]. Null associations have been reported for esophageal [21,22] and ovarian [23,24,25] cancers and coffee consumption. Coffee consumption has also been associated with increased risk of pregnancy complications [26,27] and some CVD risk factors (e.g., higher blood cholesterol levels and blood pressure [28,29,30]). While some studies have suggested a positive association [31] between coffee consumption and CVD risk, meta-analysis studies showed that there is no risk and even demonstrated benefit of moderate coffee consumption against CVD, all-cause mortality, and some cancers [32,33,34]. Furthermore, Mendelian randomization studies have shown no causal effect of coffee consumption on CVD or all-cause mortality; U-shaped associations were reported in observational studies [35,36].

Such heterogeneity in reported effects could be due to the underlying differences between studies. The definition of coffee consumption is hard to harmonize, particularly due to the wide variety of coffee types, preparation techniques, and consumption habits. For example, boiled coffee contains cafestol and kahweol, which were implicated in the cholesterol-raising effect of coffee, and these compounds could be removed by using brewed or filtered coffee [1]. 

Metabolites of caffeine and other chemical components of coffee have received much attention because they might play a role in mechanisms of health effects and could be used as biomarkers of coffee consumption. Their association with disease biomarkers is particularly interesting, as they could help identify mediators between coffee consumption and positive health effects. For example, coffee consumption was found to be inversely associated with hepatic inflammation markers [37] and hepatocellular carcinoma [38], as well as increased insulin response [39]. The mechanism behind the relationship between coffee and liver cancer is still unclear, but the inverse association with liver damage markers and the positive association with antioxidants in the coffee might be part of the answer [40,41].

Our study used an environment-wide association study (EWAS) technique to identify the metabolites, nutrients, and lifestyle factors that are associated with coffee or caffeinated beverage consumption.

## 2. Materials and Methods 

### 2.1. Study Population

We used data from the Third National Health and Nutrition Examination Survey (NHANES III) to investigate the association between coffee consumption and metabolites, nutrients, and lifestyle factors. NHANES III was a cross-sectional US survey that consisted of two phases (1988–1991 and 1991–1994). The survey used representative multistage stratified, clustered probability sampling of the US population. NHANES III data are publicly available and can be accessed online (https://wwwn.cdc.gov). Thus, institutional review board approval and oversight were not required for our study.

Based on the survey design, both phases of the NHANES III can be used independently. In our study, the first phase was used as a discovery set and the second as a replication set. Of all NHANES III participants, only data of participants at least eighteen years old at enrollment were used in the present study (*n* = 19,618). Furthermore, participants with zero weights were excluded from our analyses leading to our final study population of 17,752 participants (*n_Phase 1_* = 8825, *n_Phase 2_* = 8927).

### 2.2. Assessment of Coffee and Caffeinated Beverage Consumption

Habitual consumption of coffee, tea, and soda (including cola) was assessed using a food frequency questionnaire (FFQ) that assessed the frequency (in times per month) but not the exact amount of food consumed. The questionnaire included questions regarding consumption of caffeinated and non-caffeinated coffee, tea, and soda. However, since consumption of non-caffeinated coffee, tea and soda was very infrequent, we focused only on the consumption of caffeinated beverages, which we call "regular" in the rest of the manuscript. Total caffeinated beverage consumption expressed as number of cups per day (by assuming that the frequency reported corresponded to cups of coffee consumed during the month), was calculated by summing the consumption of regular coffee, regular tea, regular sodas (including colas), and diet sodas (including diet colas). 

### 2.3. Assessment of the Metabolites, Nutrients, and Lifestyle Factors

In our analysis, we included 245 metabolites, nutrients, and lifestyle factors (29 factors on body measurements, disease history, and medication use; 32 lifestyle factors; 80 metabolites and 104 dietary nutrients), which have previously been assessed and are publicly available (https://wwwn.cdc.gov). NHANES III participants were interviewed and underwent physical examinations in a mobile examination center. Information on some factors was obtained during the interview, while others were obtained via laboratory testing. Dietary information was collected with 24-hour dietary recall interviews, using an automated data collection instrument [42]. All NHANES III participants were asked to complete a 24-h dietary recall during their visit at the Medical Examination Center (MEC). In addition, about 5% of all adult examinees received a second replicate MEC examination that included a 24-h dietary recall; this replicate data was used to estimate within- and between-person variances for estimating nutrient intake distributions. Data collection was scheduled as such as to include all days of the week and throughout the year. Apart from reproductive factors, all factors were investigated in both men and women (Supplementary Table S1). Continuous factors were Z-score standardized. 

### 2.4. Statistical Analysis

#### 2.4.1. Discovery Phase

Using Phase 1 of the NHANES III, we assessed the association of metabolites, nutrients, and lifestyle factors with regular coffee consumption. Survey weighted linear regression models were used to examine the association of regular coffee consumption (continuous, cups per month), with continuous and dichotomous metabolites, nutrients, and lifestyle factors as explanatory variables. We then corrected for multiple testing using false discovery rate (FDR). We estimated FDR using the Benjamini–Hochberg step down method; factors at FDR < 0.05 were considered significant. The following factors were a priori considered confounders rather than predictors in this study: age, sex, race/ethnicity, education, and socioeconomic status (SES). Race/ethnicity was categorized into four groups: Non-Hispanic white, Non-Hispanic black, Mexican–American, and Other. Education was categorized as less than high school, high school equivalent, and higher than high school. SES was estimated using poverty-to-income ratio (PIR), a ratio of total family income to the official poverty threshold according to the family size. A PIR < 1 indicates that income is below the poverty threshold. We categorized PIR into four categories (PIR < 1, 1 ≤ PIR < 2, 2 ≤ PIR < 3, and PIR ≥ 3).

#### 2.4.2. Replication Phase

To replicate the results of the discovery phase we used data from Phase 2 of NHANES III. For the replication, we considered only the statistically significant factors of the discovery set (FDR < 0.05). Factors were considered relevant if their association with coffee consumption reported a *p* ≤ 0.05 in the replication set. Additionally, we examined the associations between these replicated metabolites, nutrients, and lifestyle factors and the consumption of total caffeinated beverages (continuous, cups per month).

Since the food group “soft drinks” includes not only caffeinated beverages, but also non-caffeinated soft drinks, we checked their association with the replicated metabolites, nutrients, and lifestyle factors for regular coffee consumption. We defined soft drinks as the sum of regular sodas and diet sodas and included it as a continuous variable in our model. 

In sensitivity analyses, we investigated whether the replicated metabolites, nutrients, and lifestyle factors for regular coffee consumption were similarly associated in men and women. We used survey weighted logistic regression to assess the sex-specific associations between dichotomized regular coffee consumption (at least one cup per day vs. less) and the metabolites, nutrients, and lifestyle factors replicated in the total study population. We tested for interactions with sex by adding an interaction term in all the weighted logistic regression models between coffee consumption and the metabolites, nutrients, and lifestyle factors replicated in the total study population. 

Datasets were prepared for analysis using the Statistical Analysis Software (SAS) university edition (SAS Institute, Cary, NC, US) and analyses were performed using R (version 3.2.1). Analyses were performed using the survey package including sampling weights to account for the complex survey design and survey non-response. 

## 3. Results

Among 8292 male study participants, 4111 consumed one cup of coffee or more per day (mean consumption 72.4 cups per month), and 4766 out of 9460 female participants consumed coffee at least once per day (mean consumption 62.0 cups per month; Table 1). The mean age of daily coffee drinkers was 46.6 years for men and 48.6 years for women; non-daily drinkers were younger than daily drinkers. Mean body mass index (BMI) was 26.6 kg/m^2^ for men and 26.3 kg/m^2^ for women, which was similar between daily and non-daily coffee drinkers. Daily coffee consumption was more common in Non-Hispanic whites than in Non-Hispanic blacks and in participants with higher SES as indicated by the PIR. Distribution of studied metabolites, nutrients, and lifestyle factors in men and women are provided as supplementary material (Supplementary Table S1).

### 3.1. Discovery Phase

**Figure 1:**
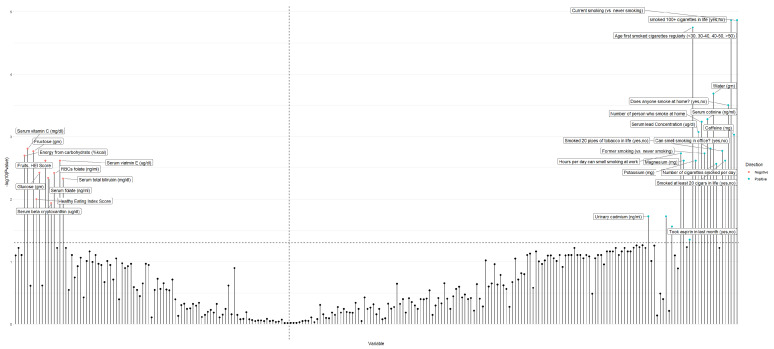
Manhattan plot depicting the associations between metabolites, nutrients, and lifestyle factors and coffee consumption in the discovery phase. Factors were ordered based on the size of effects (beta coefficient). Variable names are given only for the statistically significant metabolites, nutrients, and lifestyle factors.

We performed a systematic screening of associations between the metabolites, nutrients, and lifestyle factors with regular coffee and total caffeinated beverage consumption. Out of the 245 metabolites, nutrients, and lifestyle factors initially investigated, 32 factors were identified in the discovery set with FDR < 0.05 (Figure 1; Supplementary Table S2).

### 3.2. Replication Phase

Table 2 shows the 30 factors replicated that were significantly associated with regular coffee consumption. To summarize, coffee consumption was positively associated with both active and passive smoking, serum lead and urinary cadmium concentrations, aspirin use during the last month, and intake of magnesium, potassium, and water. It was inversely associated with folate levels in serum and red blood cells, serum concentrations of vitamin E and C and beta-cryptoxanthin, serum total bilirubin, Healthy Eating Index, and fruit intake (as assessed by the Healthy Eating Index). Most of these factors were also associated with intake of total caffeinated beverages, although not all replicated factors were statistically significant (Table 3). The association between soft drinks intake and factors replicated for regular coffee consumption were of the same direction as for regular coffee consumption (i.e., inverse association for vitamin serum levels and the Healthy Eating Index; positive association for passive smoking), but we did not see statistically significant associations with factors related to active smoking, besides former smoking.

In sex-specific analyses, all *p*-values for interactions were <0.05. However, the association between the replicated factors for regular coffee consumption identified in the replication phase and daily coffee consumption was similar in men and in women (Supplementary Table S3), with most replicated factors for regular coffee consumption showing up as statistically significant. 

## 4. Discussion

In our systematic, cross-sectional analysis of 245 metabolites, nutrients, and lifestyle factors, using a representative sample of the US population, regular coffee consumption was positively associated with smoking (both active and passive), and serum lead and urinary cadmium concentrations. In contrast, regular coffee consumption was inversely associated with serum folate, red blood cell folate levels, and the Healthy Eating Index score.

The significant association between regular coffee consumption and smoking (active and passive) could be explained by the stimulation of caffeine metabolism by smoking and thereby the higher tolerance of caffeine in smokers (i.e., they may drink more coffee or caffeine-containing beverages than non-smokers) [43,44,45]. According to a recent Mendelian randomization study, the amount of coffee consumed is unlikely to significantly affect the amount of cigarette smoking [46]. The association between coffee consumption and smoking could also be attributed to personal liability to have addictive habits [47], or a combination of all the aforementioned mechanisms. However, in this project, we are unable to judge the direction of the association between coffee consumption and smoking. 

The positive association between regular coffee consumption and serum lead or urinary cadmium concentrations in our study potentially reflects coffee contamination with heavy metals [48]. The inverse association between regular coffee consumption and serum folate or red blood cell folate concentration might indicate that coffee drinkers follow a diet less rich in fruits and vegetables compared to non-regular coffee drinkers. An inverse association between coffee consumption and folate concentration was also reported in a Norwegian study [49]. Alternately, this inverse association might be attributed to altered nutrient metabolism due to coffee consumption. 

Most factors associated with regular coffee consumption were also associated with total intake of caffeinated beverages, with the exception of fructose, glucose, magnesium, and potassium (as assessed from the 24-h dietary recall). As expected, fructose and glucose intake were positively associated with the consumption of soft drinks. Soft drinks were also inversely associated with circulating levels of folate, vitamin C and vitamin E, but not with smoking, indicating that soft drinks *per se* do not drive the association between the replicated factors for regular coffee consumption and the total caffeinated beverage consumption. A genome-wide association study, reporting no association between the genetic risk for coffee consumption and soft drink consumption, supports this finding [50].

Associations between coffee consumption and favorable metabolic profile (i.e., oxidative stress [40,51], hepatic function [37,52], or metabolic syndrome [53,54] markers) have been reported in the literature. These associations potentially explain the protective effect of coffee consumption on various diseases, including type 2 diabetes [11,12], different cancers [5,6,14,15,16,17,38] and mortality [5,6]. We acknowledge that our analysis did not detect similar significant associations. This could reflect the diversity in coffee composition (differences by type of coffee, preparation of coffee etc.) and the need for better-powered studies that are able to detect significant associations of smaller effect size.

To our knowledge, this is the first study that systematically looked at the cross-sectional association between coffee consumption and various metabolites, nutrients, and lifestyle factors simultaneously. Such associations have been the center of interest in clinical research but with reporting of only a limited group of metabolites, nutrients, or lifestyle factors in most studies. With our approach, we intended to find associations between coffee consumption and markers/indicators that represent coffee metabolites without having a specific disease in mind, thus, avoiding selective reporting and false positive results. Identifying coffee metabolites and using them as surrogates of coffee consumption or as surrogates of a certain component in coffee can help avoid misclassification bias. Our systematic approach to test associations of multiple factors with coffee or caffeinated beverage consumption allowed us to avoid selective testing of certain factors, which could be a source of bias and false positive results. Additionally, the detailed information collection at baseline allowed us to adjust for major confounders and we further replicated our results in a different dataset to verify the strong statistical associations of the results reported. 

Using NHANES III as a representative sample of the US population allowed for generalizability of our results; however, since this is a cross-sectional study, causality cannot be assessed. Some of the tested factors were self-reported, and dietary intake was mainly assessed using an FFQ that asked for frequency of consumption but not portion size. This could have resulted in misclassification not only in coffee and other beverage intake but also in confounding variables. Additionally, no information was available on the caffeine content of soft drinks. Finally, the wide variety of coffee types, cup sizes, and preparation techniques makes it challenging to accurately classify coffee consumption into subgroups or to accurately estimate caffeine intake. 

Acknowledging these limitations, we showed a systematic approach for testing the metabolites, nutrients, and lifestyle factors associated with coffee and related beverage consumption. These associations could pave the way for future studying of the benefits and risks of these beverages and their relations to diseases and mortality.

## Figures and Tables

**Table 1:** Baseline characteristics of study participants, the Third National Health and Nutrition Examination Survey (NHANES III).

Frequency of Coffee Consumption	Men		Women	
	<1 cup/day	≥1 cup/day	<1 cup/day	≥1 cup/day
No. study participants ^1^ (%)	4163 (50.31)	4111 (49.69)	5182 (54.86)	4264 (45.14)
Age, mean (SE), years	38.64 (0.49)	46.61 (0.51)	41.15 (0.53)	48.62 (0.56)
BMI, mean (SE), kg/m^2^	26.32 (0.14)	26.59 (0.13)	26.36 (0.24)	26.31 (0.13)
Race/Ethnicity, %				
Non-Hispanic White	69.52	81.99	71.16	80.84
Non-Hispanic Black	15.45	5.79	15.48	7.72
Mexican–American	6.27	5.11	4.99	4.44
Other	8.76	7.11	8.36	7.00
Education, %				
Less than high school	9.45	13.25	10.27	12.51
High school equivalent	45.82	45.76	48.90	51.99
Higher than high school	44.73	40.99	40.83	35.50
PIR category, %				
PIR < 1	12.18	10.24	17.16	12.40
1 ≤ PIR < 2	21.83	18.47	22.36	22.27
2 ≤ PIR < 3	21.98	21.76	20.81	19.70
PIR ≥ 3	44.01	49.53	39.67	45.63

^1^ Number of study participants is unweighted. Abbreviations: BMI, body mass index; SE, standard error; PIR, poverty-to-income ratio.

**Table 2:** Replicated metabolites, nutrients, and lifestyle factors associated with regular coffee consumption (assessed as a continuous variable).

Factor	No. ^1^	Coefficient	SE	*p*-Value
Water (gm) ^2^	7681	13.01	2.42	1.67 × 10^−4^
Caffeine (mg) ^2^	7681	21.57	2.70	3.78 × 10^−6^
Smoked 100+ cigarettes in life (yes, no)	8138	21.58	2.26	6.01 × 10^−7^
Age first smoked cigarettes regularly (<30, 30–40, 40–50, >50)	8002	9.68	1.06	9.38 × 10^−7^
Number of cigarettes smoked per day	1801	10.36	3.25	7.83 × 10^−3^
Former smoking (vs. never smoking)	6215	10.49	1.73	5.69 × 10^−5^
Current smoking (vs. never smoking)	6248	34.79	4.09	1.97 × 10^−6^
Smoked at least 20 cigars in life (yes, no)	8135	14.40	5.16	1.63 × 10^−2^
Smoked 20 pipes of tobacco in life (yes, no)	8135	19.34	6.90	1.60 × 10^−2^
Serum cotinine (ng/mL)	7603	12.25	1.78	1.68 × 10^−5^
Does anyone smoke at home? (yes, no)	8138	21.34	2.34	9.40 × 10^−7^
Can smell smoking in office? (yes, no)	4467	15.86	4.44	3.86 × 10^−3^
Number of persons who smoke at home	8138	11.24	1.66	1.99 × 10^−5^
Hours per day can smell smoking at work	4467	8.30	2.48	5.79 × 10^−3^
Took aspirin in last month (yes, no)	8127	5.46	2.43	4.47 × 10^−2^
Serum Lead Concentration (ug/dL)	7866	8.25	1.71	4.23 × 10^−4^
Urinary cadmium (ng/mL)	7783	4.04	1.52	2.11 × 10^−2^
Serum total bilirubin (mg/dL)	7771	−4.73	1.29	3.27 × 10^−3^
Serum beta cryptoxanthin (ug/dL)	7791	−4.62	0.88	2.16 × 10^−4^
RBCs folate (ng/mL)	7632	−6.04	0.56	1.70 × 10^−7^
Serum folate (ng/mL)	7824	−4.03	1.27	8.08 × 10^−3^
Serum vitamin C (mg/dL)	7372	−4.21	0.72	7.57 × 10^−5^
Serum vitamin E (ug/dL)	7792	−3.25	0.95	4.94 × 10^−3^
Fruits, Healthy Eating Index Score ^2^	7681	−4.06	1.07	2.62 × 10^−3^
Healthy Eating Index Score ^2^	7681	−5.03	0.99	2.82 × 10^−4^
Energy from carbohydrate (%kcal) ^2^	7681	−5.17	1.50	4.86 × 10^−3^
Fructose (gm) ^2^	7681	−5.28	0.82	3.22 × 10^−5^
Glucose (gm) ^2^	7681	−5.09	0.99	2.53 × 10^−4^
Magnesium (mg) ^2^	7681	4.35	1.58	1.75 × 10^−2^
Potassium (mg) ^2^	7681	5.45	1.53	3.89 × 10^−3^

^1^ Number of study participants is unweighted. ^2^ Assessed from 24-hour dietary recall interviews. Abbreviations: gm, gram; mg, milligram; RBCs, red blood cell; SE, standard error.

**Table 3:** Replicated metabolites, nutrients, and lifestyle factors of regular coffee consumption and their association with consumption of caffeinated beverages and soft drinks (continuous).

	Caffeinated Beverages			Soft Drinks		
Factor	Coefficient	SE	*p*-Value	Coefficient	SE	*p*-Value
Water (gm) ^1^	19.64	2.07	6.32 × 10^−7^	3.46	0.82	1.20 × 10^−3^
Caffeine (mg) ^1^	-- ^2^			--		
Smoked 100 + cigarettes in life (yes, no)	26.09	3.01	1.62 × 10^−6^	--		
Age first smoked cigarettes regularly (<30, 30–40, 40–50, > 50)	11.73	1.48	4.19 × 10^−6^	--		
Number of cigarettes smoked per day	17.22	2.99	8.90 × 10^−5^	--		
Former smoking (vs. never smoking)	11.29	2.83	1.78 × 10^−3^	5.61	1.60	4.27 × 10^−3^
Current smoking (vs. never smoking)	41.76	4.81	1.60 × 10^−6^	--		
Smoked at least 20 cigars in life (yes, no)	19.44	7.99	3.16 × 10^−2^	--		
Smoked 20 pipes of tobacco in life (yes, no)	23.27	7.62	1.00 × 10^−2^	--		
Serum cotinine (ng/mL)	16.01	2.07	5.29 × 10^−6^	2.63	0.58	7.08 × 10^−4^
Does anyone smoke at home? (yes, no)	29.11	3.25	1.16 × 10^−6^	4.61	1.27	3.51 × 10^−3^
Can smell smoking in office? (yes, no)	22.76	5.93	2.35 × 10^−3^	--		
Number of persons who smoke at home	14.86	2.49	6.53 × 10^−5^	2.40	0.73	6.34 × 10^−3^
Hours per day can smell smoking at work	13.27	2.78	4.53 × 10^−4^	--		
Took aspirin in last month (yes, no)	--			--		
Serum Lead Concentration (ug/dL)	8.09	1.92	1.20 × 10^−3^	--		
Urinary cadmium (ng/mL)	4.99	1.15	9.66 × 10^−4^	--		
Serum total bilirubin (mg/dL)	−6.58	1.63	1.61 × 10^−3^	--		
Serum beta cryptoxanthin (ug/dL)	−9.56	1.46	2.82 × 10^−5^	−3.43	0.54	3.64 × 10^−5^
RBCs folate (ng/mL)	−9.99	1.02	4.55 × 10^−7^	−2.49	0.66	2.77 × 10^−3^
Serum folate (ng/mL)	−7.14	1.35	1.94 × 10^−4^	−2.39	0.48	3.24 × 10^−4^
Serum vitamin C (mg/dL)	−10.37	1.37	6.53 × 10^−6^	−4.36	0.75	8.85 × 10^−5^
Serum vitamin E (ug/dL)	−3.96	1.48	1.99 × 10^−2^	--		
Fruits, Healthy Eating Index Score ^1^	−7.86	1.60	3.57 × 10^−4^	−2.66	0.6	8.31 × 10^−4^
Healthy Eating Index Score ^1^	−8.87	1.70	2.19 × 10^−4^	−2.97	0.63	5.13 × 10^−4^
Energy from carbohydrate (%kcal) ^1^	−4.45	1.64	1.90 × 10^−2^	--		
Fructose (gm) ^1^	--			2.74	0.78	4.27 × 10^−3^
Glucose (gm) ^1^	--			2.76	0.83	6.14 × 10^−3^
Magnesium (mg) ^1^	--			--		
Potassium (mg) ^1^	--			--		

^1^ Assessed from 24-h dietary recall interviews. ^2^ The dashed lines represent coffee-replicated factors that failed replication in either soft drinks or caffeinated beverages consumption. Coefficients are only shown for replicated results. Abbreviations: gm, gram; mg, milligram; RBCs, red blood cell; SE, standard error.

## Supplementary Materials

The following are available online at https://www.mdpi.com/2072-6643/12/5/1470/s1: Table S1. Survey weighted descriptive statistics of metabolites, nutrients, and lifestyle factors in the Third National Health and Nutrition Examination Survey (NHANES III). Survey weighted means and standard errors (SE) are reported for continuous variables while proportions are reported for categorical variables; Table S2. Associations between metabolites, nutrients, and lifestyle factors and regular coffee consumption in the discovery set. All models were adjusted for age, race/ethnicity, education, and poverty-to-income ratio (PIR). Benjamini–Hochberg adjusted *p*-values for false discovery rate (FDR) < 5% are shown; Table S3. Validated metabolites, nutrients, and lifestyle factors of regular coffee consumption and their association with coffee consumption (continuous) stratified by sex.
