# Supplementary material for: Metabolites, Nutrients, and Lifestyle Factors in Relation to Coffee Consumption: An Environment-Wide Association Study
Source: Nutrients. 2020 May 19;12(5):1470. doi: 10.3390/nu12051470 (PMC7284649; doi:10.3390/nu12051470)
Supplement: Supplementary file 1 [file nutrients-12-01470-s001.pdf]

Table S1: Survey weighted descriptive statistics of metabolites, nutrients and lifestyle factors in NHANES III. Survey weighted means and standard errors (SE) are reported for continuous variables while proportions are reported for categorical variables.

Variable	Full Name	Men		Women	
		N	Weighted mean (SE) or proportion	N	Weighted mean (SE) or proportion
a_carot	Serum alpha carotene (ug/dL)	7,802	4.14 (0.09)	8,827	5.28 (0.12)
a_toco_g	Alpha-tocopherol (mg)	7,802	9.31 (0.19)	8,887	6.87 (0.18)
age_epil	Age first took hormone pills	0	N/A	1,211	44.11 (0.45)
age_pill	Age first took BC pills	0	N/A	4,713	21.45 (0.11)
age_stop	Age last smoked cigarettes regularly	2,655	38.04 (0.47)	1,560	38.58 (0.62)
agebaby	Age at first child birth	0	N/A	7,020	22.25 (0.1)
alan_g	Alanine (gm)	7,802	4.73 (0.07)	8,887	3.1 (0.04)
alc_mo	Alcohol consumption per month	8,260	12.24 (0.45)	9,448	5.1 (0.31)
alco_g	Alcohol (gm)	7,802	15.05 (0.8)	8,887	5.52 (0.48)
amp	Serum albumin (g/dL)	7,719	4.3 (0.02)	8,777	4.08 (0.02)
anprot_g	Animal Protein (gm)	7,802	68.83 (1.14)	8,887	44.61 (0.63)
apoa	Serum apolipoprotein A1 (mg/dL)	4,090	135.71 (0.88)	4,039	150.29 (1.06)
apob	Serum apolipoprotein B (mg/dL)	4,101	105.44 (0.97)	4,049	102.18 (0.9)
appsi	ALP_Serum alkaline phosphatase (U/L)	7,718	85.58 (0.7)	8,776	80.02 (0.87)
argin_g	Arginine (gm)	7,802	5.43 (0.07)	8,887	3.56 (0.04)
ash_g	Ash (gm)	7,802	21.51 (0.2)	8,887	14.99 (0.14)
aspac_g	Aspartic Acid (gm)	7,802	8.42 (0.11)	8,887	5.58 (0.06)
aspar_g	Aspartame (mg)	7,802	56.29 (3.23)	8,887	69.89 (3.19)
aspi_mo	Amount of aspirin taken past month	2,783	13.49 (0.74)	2,844	13.59 (0.63)
aspirin	Took aspirin in last month (yes, no)	8,243	59.93, 40.07	9,407	64.42, 35.58
aspsi	AST Aspartate aminotransferase (U/L)	7,719	23.37 (0.25)	8,777	19.61 (0.2)
atpsi	ALT Alanine aminotransferase (U/L)	7,719	21.07 (0.55)	8,777	14.54 (0.31)
b_carot	Serum beta carotene (ug/dL)	7,802	16.91 (0.36)	8,827	22.52 (0.37)
b_crypto	Serum beta cryptoxanthin (ug/dL)	7,802	8.74 (0.14)	8,826	9.51 (0.19)
b_toco_g	Beta-tocopherol (mg)	7,802	0.33 (0.01)	8,887	0.24 (0.01)
bcaro_g	Beta-carotene (mcg)	7,802	3014.57 (95.51)	8,887	2662.15 (72.57)
bcpill	History of birth control pills	0	N/A	8,903	38.38, 61.62
bmi	Body mass index (BMI)	8,271	26.46 (0.11)	9,418	26.34 (0.15)
br_feed	Breat feeding (yes,no)	0	N/A	7,032	45.34, 54.66
breastca	History of breast cancer (yes, no)	616	99.96, 0.04	714	81.74, 18.26
bup	Serum blood urea nitrogen (mg/dL)	7,719	15.28 (0.12)	8,777	13.33 (0.13)
ca_norm	Serum normalized calcium (mmol/L)	6,902	1.24 (0)	7,826	1.23 (0)
ca_tot	Serum total calcium (mmol/L)	7,636	2.31 (0)	8,620	2.3 (0)
cadmium	Urinary cadmium (ng/mL)	7,909	0.64 (0.02)	8,931	0.66 (0.02)
cafe_g	Caffeine (mg)	7,802	291.69 (11.55)	8,887	212.27 (5.55)
calc_g	Calcium (mg)	7,802	959.24 (15.54)	8,887	696.03 (9.76)
cancer	History of cancer (yes, no)	8,291	93.78, 6.22	9,459	91.31, 8.69
carb_g	Total carbohydrates (gm)	7,802	317.27 (3.59)	8,887	222.73 (2.18)
cep	Serum creatinine (mg/dL)	7,718	1.18 (0)	8,777	0.97 (0)
cfiber_g	Crude Fiber (gm)	7,802	5.12 (0.06)	8,887	3.84 (0.04)
chewsnuf	Chewing tobacco or snuff (yes, no)	8,292	81.44, 18.56	9,458	97.97, 2.03
cho_hei	Cholesterol, HEI Score	7,802	6.63 (0.11)	8,887	8.37 (0.07)
chol_g	Cholesterol (mg)	7,802	353.75 (7.18)	8,887	224.7 (4.26)

cig_100	Smoked 100+ cigarettes in life (yes, no)	8,292	37.32, 62.68	9,460	55.12, 44.88
cig_age	Age first smoked cigarettes regularly (<30, 30-40, 40-50, >50)	8,106	10.32 (0.15)	9,333	8.22 (0.2)
cig_day	Number of cigarettes smoked per day	2,325	20.88 (0.52)	1,873	18.18 (0.43)
cig_dmax	Number of cigarettes per day when smoking most	857	35.15 (1.13)	646	26.68 (1.01)
cig_form	Former smoking (vs never smoking)	5,785	54.85, 45.15	7,465	73.62, 26.38
cig_now	Current smoking (vs never smoking)	5,586	53.88, 46.12	7,877	68.68, 31.32
cigar_20	Smoked at least 20 cigars in life (yes, no)	8,291	74.13, 25.87	9,456	99.33, 0.67
cop	Serum cotinine (ng/mL)	7,630	95.35 (3.16)	8,567	61.58 (2.54)
copp_g	Copper (mg)	7,802	1.65 (0.02)	8,887	1.17 (0.02)
crp	Serum C-reactive protein (mg/dL)	7,776	0.36 (0.01)	8,821	0.46 (0.01)
cvd	Cardiovascular disease (yes,no)	8,259	98.76, 1.24	9,412	99.23, 0.77
cyst_g	Cystine (gm)	7,802	1.29 (0.02)	8,887	0.86 (0.01)
d_toco_g	Delta-tocopherol (mg)	7,802	5.25 (0.12)	8,887	3.72 (0.06)
dai_hei	Dairy, HEI Score	7,802	6.78 (0.07)	8,887	6.03 (0.07)
diastole	Diastolic blood pressure	7,971	76.84 (0.28)	9,073	72.68 (0.27)
dm	Diabetes mellitus (yes, no)	8,285	95.33, 4.67	9,446	94.06, 5.94
dmdrug	Under diabetes treatment (yes, no)	3,144	92.72, 7.28	4,828	94.93, 5.07
dwp	Platelet distribution width (%)	7,823	16.52 (0.02)	8,883	16.39 (0.02)
ecream	History of female hormonal therapy in the form of (vaginal cream, suppository or injection)	0	N/A	4,133	89.9, 10.1
epatch	History of female hormonal patch	0	N/A	4,158	95.88, 4.12
epill	History of estrogen or female hormones other than BC pills	0	N/A	4,448	64.78, 35.22
epp	Erythrocyte protoporphyrin (ug/dL)	7,926	43.69 (0.38)	8,988	55.59 (0.48)
fat_hei	Fats, HEI Score	7,802	6.34 (0.11)	8,887	6.51 (0.07)
fbp	Plasma fibrinogen (mg/dL)	4,461	298.77 (2.99)	4,889	310.99 (2.86)
fcig_day	Number of cigarettes per day before quitting	2,542	24.64 (0.5)	1,481	17.64 (0.5)
fiber_g	Dietary fiber (gm)	7,802	20.05 (0.18)	8,887	14.69 (0.17)
fola_g	Folic acid (mcg)	7,802	321.16 (3.64)	8,887	233.97 (3.37)
folate_r	RBCs folate (ng/mL)	7,737	188.84 (2.58)	8,725	206.42 (3.17)
folate_s	Serum folate (ng/mL)	7,872	6.14 (0.15)	8,922	7.46 (0.19)
frp	Serum ferritin (ng/mL)	7,870	175.42 (3.21)	8,917	81.34 (1.73)
fru_hei	Fruits, HEI Score	7,802	3.28 (0.08)	8,887	3.98 (0.09)
fruc_g	Fructose (gm)	7,800	32.48 (0.66)	8,887	23.08 (0.37)
fshiu	Serum FSH (IU/L)	0	N/A	3,122	29.06 (0.89)
g_toco_g	Gamma-tocopherol (mg)	7,802	21.59 (0.39)	8,887	15.42 (0.23)
gala_g	Galactose (gm)	7,802	0.19 (0.01)	8,887	0.19 (0.01)
gbp	Serum globulin (g/dL)	5,738	3.1 (0.02)	6,617	3.21 (0.02)
ggpsi	GGT_Gamma glutamyl transferase (U/L)	5,938	35.6 (1.07)	6,862	23.56 (0.62)
gluc_g	Glucose (gm)	7,802	33.91 (0.69)	8,887	23.66 (0.35)
glucose	Serum glucose (mg/dL)	7,717	98.14 (0.62)	8,775	95.48 (0.93)
glutac_g	Glutamic Acid (gm)	7,802	19.22 (0.21)	8,887	12.99 (0.13)
glyc_g	Glycine (gm)	7,802	4.21 (0.06)	8,887	2.73 (0.03)
gra_hei	Grains, HEI Score	7,802	6.87 (0.07)	8,887	6.25 (0.06)
granp	Granulocyte percent	7,740	61.96 (0.2)	8,736	62.2 (0.21)
grp	Granulocyte number	7,740	4.55 (0.04)	8,736	4.61 (0.04)

ha_ab	Serum Hepatitis A Antibody	7,625	65.19, 34.81	8,607	62.85, 37.15
hba1c	Glycated Hemoglobin	7,926	5.38 (0.02)	8,986	5.32 (0.03)
hbc_ab	Serum Hepatitis B Core Antibody	7,629	92.8, 7.2	8,610	94.84, 5.16
hbs_ab	Serum Hepatitis B Surface Antibody	391	21.79, 78.21	321	16.07, 83.93
hbs_ag	Serum Hepatitis B Surface Antigen	109	53.69, 46.31	65	55.96, 44.04
hc_ab	Serum Hepatitis C Antibody	7,608	96.93, 3.07	8,588	98.6, 1.4
hctp	Hematocrit (%)	7,865	44.55 (0.08)	8,928	39.48 (0.08)
hdl	HDL cholesterol (mg/dL)	7,787	45.78 (0.36)	8,828	55.21 (0.4)
heiscore	Healthy Eating Index Score (HEI)	7,802	62.27 (0.34)	8,887	64.6 (0.31)
hgp	Hemoglobin (g/dl)	7,865	15.11 (0.03)	8,930	13.28 (0.03)
histi_g	Histidine (gm)	7,802	2.82 (0.04)	8,887	1.85 (0.02)
homocyst	Serum Homocysteine	3,257	10.75 (0.18)	4,201	8.89 (0.14)
hppcag	H Pylori CagA Seropositivity	3,749	74.81, 25.19	3,716	76.38, 23.62
hpylori	Serum Helicobacter Pylori Antibody	3,910	67.05, 32.95	3,902	68.42, 31.58
hrt	History of hormone replacement therapy	0	N/A	4,105	57.5, 42.5
hsv1	Serum Herpes I Antibody	5,511	35.3, 64.7	5,710	28.25, 71.75
hsv2	Serum Herpes II Antibody	5,494	80.98, 19.02	5,692	72.06, 27.94
ht	Hypertension (yes, no)	8,272	78.42, 21.58	9,446	75.37, 24.63
htdrug	Under hypertension treatment (yes, no)	3,140	77.08, 22.92	4,824	81.13, 18.87
ifiber_g	Water insoluble dietary fiber (gm)	7,802	12.91 (0.14)	8,887	9.56 (0.12)
igp_bp3	Insulin like growth factor binding protein-3 (ng/ml)	2,742	4389.65 (37.61)	3,318	4538.85 (32.12)
igp_i	Insulin like growth factor-I (ng/ml)	2,742	285.58 (3)	3,317	259.56 (4.14)
implant	Subcutaneous implant NORPLANT	0	N/A	2,125	98.79, 1.21
insulin	Serum Insulin	7,394	11.23 (0.24)	8,360	11.16 (0.37)
iodine	Urinary iodine (ug/dL)	7,849	22.6 (1.38)	8,810	24.55 (4)
iron_g	Iron (mg)	7,802	18.64 (0.2)	8,887	13.11 (0.2)
isoleu_g	Isoleucine (gm)	7,802	4.5 (0.06)	8,887	3.03 (0.03)
kcal	Calories (kcal)	7,802	2673.32 (27.25)	8,887	1784.81 (14.8)
l1p	Serum latex antibody (IU/mL)	2,704	0.69 (0.05)	2,646	0.42 (0.05)
lact_g	Lactose (gm)	7,802	16.61 (0.46)	8,887	12.53 (0.28)
lbdvidms	Vitamin D (nmol/L)	7,762	64.97 (0.61)	8,807	58.92 (0.67)
ldl	LDL cholesterol (mg/dL)	3,268	128.95 (0.9)	3,773	124.54 (1.25)
ldpsi	LDH_Serum lactate dehydrogenase (U/L)	7,718	158.45 (2.03)	8,777	156.11 (2.13)
lead	Serum Lead Concentration (ug/dL)	7,956	4.4 (0.13)	9,002	2.7 (0.08)
lep	Serum leptin	2,937	6.01 (0.16)	3,478	16.58 (0.42)
leuc_g	Leucine (gm)	7,802	7.74 (0.1)	8,887	5.16 (0.06)
lhiu	Serum LH (IU/L)	0	N/A	3,120	12.2 (0.35)
lipiddrug	Under lipid lowering treatment (yes, no)	3,140	98.24, 1.76	4,824	98.86, 1.14
lipop	Serum lipoprotein A (mg/dL)	3,725	20.82 (1.06)	4,809	23.75 (1.01)
lmp	Lymphocyte number	7,865	2.25 (0.02)	8,928	2.27 (0.01)
lut_zea	Serum lutein zeaxanthin (ug/dL)	7,802	21.47 (0.3)	8,827	21.93 (0.33)
lycopene	Serum lycopene (ug/dL)	7,802	24.66 (0.35)	8,827	22.33 (0.28)
lymp	Lymphocyte percent	7,865	31.7 (0.21)	8,928	32.21 (0.2)
lysin_g	Lysine(gm)	7,802	6.79 (0.1)	8,887	4.47 (0.06)
magn_g	Magnesium (mg)	7,802	352.23 (2.99)	8,887	249.2 (3.05)
malt_g	Maltose (gm)	7,802	3.8 (0.11)	8,887	2.49 (0.07)
mcpsi	MCH	7,865	30.56 (0.05)	8,928	30.19 (0.05)
mea_hei	Meats, HEI Score	7,802	7.68 (0.07)	8,887	6.47 (0.06)

meet_yr	Meetings with friends, relatives or neighbors at least once a week (yes, no)	8,290	18.1, 81.9	9,459	15.36, 84.64
menarche	Age of menarche	0	N/A	8,734	12.78 (0.03)
menopause	Menopause (0=premenopause, 1=perimenopause, 2=postmenopause)	0	N/A	8,913	55.62, 5.5, 38.88
meth_g	Methionine (gm)	7,802	2.27 (0.03)	8,887	1.51 (0.02)
mfa141_g	MFA 14:1 (gm)	7,802	0.08 (0)	8,887	0.04 (0)
mfa161_g	MFA 16:1 (gm)	7,802	2 (0.04)	8,887	1.22 (0.02)
mfa181_g	Oleic acid (MFA 18:1) (gm)	7,802	37.64 (0.63)	8,887	24.42 (0.26)
mfa201_g	MFA 20:1 (gm)	7,802	0.17 (0.01)	8,887	0.11 (0)
mfa221_g	MFA 22:1 (gm)	7,802	0.02 (0)	8,887	0.01 (0)
mhp	MCHC	7,865	33.91 (0.04)	8,928	33.63 (0.04)
monop	Mononuclear percent	7,740	6.3 (0.1)	8,736	5.57 (0.09)
mop	Mononuclear number	7,740	0.45 (0.01)	8,736	0.4 (0.01)
mufat_g	Total monounsaturated fatty acids (gm)	7,802	40.31 (0.67)	8,887	26.08 (0.28)
mvpsi	MCV	7,865	90.12 (0.15)	8,929	89.72 (0.14)
niac_g	Niacin (mg)	7,802	29.22 (0.25)	8,887	19.62 (0.23)
now_epil	<1 month since last took hormone pills	0	N/A	4,106	86.19, 13.81
now_pill	<1 month since last took BC pills	0	N/A	4,704	81.25, 18.75
nsa_mo	Amount of NSAID takes past month	1,154	9.15 (0.81)	2,165	10.24 (0.64)
nsaid	Took NSAID in last month (yes, no)	8,248	80.52, 19.48	9,409	70.62, 29.38
ospsi	Serum osmolality (mmol/Kg)	5,738	280.3 (0.7)	6,617	278.24 (0.7)
oxal_g	Oxalic Acid (mg)	7,802	369.5 (5.32)	8,887	283.63 (3.76)
p_alc	Energy from alcohol (%kcal)	7,802	3.62 (0.17)	8,887	2.02 (0.15)
p_carb	Energy from carbohydrate (%kcal)	7,802	48.23 (0.36)	8,887	50.82 (0.28)
p_msfat	Energy from monosaturated fat (%kcal)	7,802	13.26 (0.13)	8,887	12.7 (0.08)
p_prot	Energy from protein (%kcal)	7,802	15.15 (0.1)	8,887	15.21 (0.09)
p_psfat	Energy from polysaturated fat (%kcal)	7,802	6.53 (0.06)	8,887	6.81 (0.07)
p_sfat	Energy from saturated fat (%kcal)	7,802	11.8 (0.09)	8,887	11.43 (0.1)
p_tfat	Energy from total fat (%kcal)	7,802	34.23 (0.26)	8,887	33.5 (0.22)
pantho_g	Pantothenic acid (mg)	7,802	5.7 (0.07)	8,887	4.07 (0.08)
parity	Parity (0=nulliparous, 1=primiparous, >2=multiparous)	0	N/A	7,516	8.32, 20.51, 71.17
pct	Took paracetamol in last month (yes, no)	8,246	67.09, 32.91	9,416	55.12, 44.88
pct_mo	Amount of paracetamol takes past month	2,455	8.19 (0.46)	3,997	11.23 (0.6)
pect_g	Pectin (gm)	7,802	2.14 (0.03)	8,886	1.55 (0.02)
pfa182_g	Linoleic acid (PFA 18:2) (gm)	7,802	17.56 (0.3)	8,887	12.44 (0.19)
pfa183_g	Linolenic acid (PFA 18:3) (gm)	7,802	1.65 (0.03)	8,887	1.17 (0.02)
pfa184_g	PFA 18:4 (gm)	7,802	0 (0)	8,887	0 (0)
pfa204_g	PFA 20:4 (gm)	7,802	0.17 (0)	8,887	0.11 (0)
pfa205_g	PFA 20:5 (gm)	7,802	0.05 (0)	8,887	0.04 (0)
pfa225_g	PFA 22:5 (gm)	7,802	0.02 (0)	8,887	0.01 (0)
pfa226_g	PFA 22:6 (gm)	7,802	0.1 (0.01)	8,887	0.07 (0.01)
phen_g	Phenylalanine (gm)	7,802	4.32 (0.05)	8,887	2.89 (0.03)
phone_wk	Daily phone calls with friends, relatives or neighbors (yes, no)	6,716	52.96, 47.04	8,543	32.75, 67.25
phos_g	Phosphorous (mg)	7,802	1555.71 (16.2)	8,887	1066.96 (12.02)
phosp	Serum phosphorus (mg/dL)	7,719	3.37 (0.01)	8,777	3.53 (0.01)
phyt_g	Phytic Acid (mg)	7,802	782.96 (13.41)	8,887	558.12 (8.94)

pipe_20	Smoked 20 pipes of tobacco in life (yes, no)	8,288	79.59, 20.41	9,454	99.65, 0.35
pltp	Platelet Count	7,863	257.77 (2.38)	8,928	282.28 (2.21)
pots_g	Potassium (mg)	7,802	3227.19 (27.07)	8,887	2324.33 (25.71)
pregnant	Pregnancy (yes,no)	0	N/A	4,238	94.35, 5.65
proli_g	Proline (gm)	7,802	6.46 (0.07)	8,887	4.38 (0.04)
prot_g	Protein (gm)	7,802	99.24 (1.21)	8,887	66.03 (0.7)
pufat_g	Total polyunsaturated fatty acids (gm)	7,802	19.69 (0.33)	8,887	13.93 (0.21)
pvpsi	Mean platelet volume	7,865	8.38 (0.03)	8,930	8.46 (0.03)
pxp	Serum transferrin saturation (%)	7,866	28.93 (0.24)	8,909	23.9 (0.25)
rcp	RBCs count	7,865	4.96 (0.01)	8,928	4.41 (0.01)
retino_g	Retinol (mcg)	7,802	578.93 (18.14)	8,887	432.54 (12.6)
rfp	Serum rheumatoid factor antibody	2,558	46.32 (9.91)	2,713	47.26 (8.96)
ribo_g	Riboflavin (mg)	7,802	2.39 (0.03)	8,887	1.65 (0.02)
rubella	Serum Rubella Antibody IU	7,688	110.87 (2.53)	8,701	101.06 (1.94)
rubella_cat	Serum Rubella Categorical	7,548	7.2, 92.8	8,470	7.25, 92.75
rwp	Red cell distribution width (%)	7,865	12.89 (0.02)	8,930	13.01 (0.02)
sacch_g	Saccharin (mg)	7,802	7.08 (0.92)	8,887	9.43 (0.62)
satfat_g	Total saturated fatty acids (gm)	7,802	35.89 (0.61)	8,887	23.48 (0.29)
scl	Serum chloride (mmol/L)	7,719	104.35 (0.2)	8,777	104.65 (0.2)
sele_g	Selenium (mcg)	7,802	145.68 (1.77)	8,887	99.02 (1.46)
selenium	Serum selenium (ng/mL)	7,650	126.88 (0.99)	8,734	123.82 (0.89)
serin_g	Serine (gm)	7,802	4.43 (0.05)	8,887	2.98 (0.03)
sfa_hei	Saturated Fat, HEI Score	7,802	6.25 (0.11)	8,887	6.39 (0.09)
sfa100_g	SFA 10:0 (gm)	7,802	0.58 (0.02)	8,887	0.39 (0.01)
sfa120_g	SFA 12:0 (gm)	7,802	1.33 (0.05)	8,887	0.89 (0.02)
sfa140_g	SFA 14:0 (gm)	7,802	3.06 (0.07)	8,887	2.03 (0.03)
sfa160_g	SFA 16:0 (gm)	7,802	19.16 (0.31)	8,887	12.48 (0.15)
sfa170_g	SFA 17:0 (gm)	7,802	0 (0)	8,887	0 (0)
sfa180_g	SFA 18:0 (gm)	7,802	9.62 (0.17)	8,887	6.21 (0.07)
sfa200_g	SFA 20:0 (gm)	7,802	0.03 (0)	8,887	0.02 (0)
sfa220_g	SFA 22:0 (gm)	7,802	0.04 (0)	8,887	0.02 (0)
sfa40_g	SFA 4:0 (gm)	7,802	0.63 (0.02)	8,887	0.44 (0.01)
sfa60_g	SFA 6:0 (gm)	7,802	0.31 (0.01)	8,887	0.22 (0.01)
sfa80_g	SFA 8:0 (gm)	7,802	0.31 (0.01)	8,887	0.21 (0)
sfe	Serum iron (ug/dL)	7,885	98.7 (0.71)	8,938	84.66 (0.88)
sfiber_g	Water soluble dietary fiber (gm)	7,802	6.94 (0.05)	8,887	5 (0.05)
shco3	Serum bicarbonate (mmol/L)	7,718	28.68 (0.23)	8,776	27.6 (0.24)
shs	Passive smoking (yes,no)	3,079	13.12, 86.88	5,882	24.43, 75.57
shs_cop	Passive smoking by COP (yes, no)	2,830	11.03, 88.97	5,319	20.39, 79.61
shs_home	Does anyone smoke at home? (yes, no)	8,282	61.59, 38.41	9,450	65.56, 34.44
shs_work	Can smell smoking in office? (yes, no)	5,124	52.26, 47.74	4,474	66.02, 33.98
sk	Serum potassium (mmol/L)	7,719	4.11 (0.01)	8,777	4.02 (0.01)
smoke_hm	Number of person who smoke at home	8,282	0.6 (0.03)	9,450	0.52 (0.02)
smoke_ofc	Hours per day can smell smoking at work	5,124	2.44 (0.13)	4,474	1.64 (0.12)
sna	Serum sodium (mmol/L)	7,719	141.64 (0.1)	8,777	140.98 (0.11)
sod_hei	Sodium, HEI Score	7,802	4.41 (0.1)	8,887	7.13 (0.08)
sodi_g	Sodium (mg)	7,802	4326.55 (48.4)	8,887	2940.17 (31.94)
ssb2m	Beta-2 microglobulin (mg/L)	3,391	2.12 (0.04)	3,750	2.17 (0.03)
ssbtp	Beta-trace protein (mg/L)	3,388	0.65 (0.01)	3,748	0.63 (0.01)

sscystat	Cystatin C (mg/L)	3,381	0.99 (0.01)	3,735	0.96 (0.01)
star_g	Starch (gm)	7,802	137.17 (1.43)	8,887	93.7 (1.22)
sucr_g	Sucrose (gm)	7,802	64.05 (1.81)	8,887	45.58 (0.77)
sum_ret	Serum sum retinyl esters (ug/dL)	7,802	5.85 (0.09)	8,827	5.92 (0.1)
surgimenop	Surgical menopause	0	N/A	9,460	82.02, 17.98
systole	Systolic blood pressure	7,971	125.72 (0.4)	9,073	121.09 (0.53)
t4p	Serum thyroxine (ug/mL)	7,392	8.4 (0.06)	8,554	9.12 (0.07)
tap	Serum antithyroglobulin antibody (U/mL)	7,471	3.66 (0.56)	8,485	8.99 (1.47)
tbp	Serum total bilirubin (mg/dL)	7,719	0.72 (0.01)	8,777	0.53 (0.01)
tcp	Serum cholesterol (mg/dL)	7,847	200.73 (0.88)	8,885	205.16 (0.93)
tetanus	Serum Tetanus Antibody	6,698	1.17 (0.04)	7,366	0.84 (0.03)
tgp	Serum triglycerides (mg/dL)	7,831	154.49 (2.83)	8,864	131.44 (2.54)
thia_g	Thiamin (mg)	7,802	2.11 (0.02)	8,887	1.46 (0.02)
thp	Serum TSH (uU/mL)	7,471	1.89 (0.06)	8,485	2.26 (0.09)
thre_g	Threonine (gm)	7,802	1.16 (0.01)	8,887	0.78 (0.01)
tip	Serum TIBC (ug/dL)	7,876	346.67 (2.06)	8,918	363.99 (1.96)
tmp	Serum antimicrosomal antibody (U/mL)	7,471	4.9 (0.82)	8,485	12.56 (0.81)
totfat_g	Total fats (gm)	7,802	103.76 (1.62)	8,887	68.59 (0.77)
toxo	Serum Toxoplasmosis Antibody	7,277	23.6 (1.35)	8,247	21.83 (1.08)
tpp	Serum total protein (g/dL)	7,719	7.36 (0.02)	8,777	7.25 (0.02)
tryp_g	Tryptophan (gm)	7,802	1.16 (0.01)	8,887	0.78 (0.01)
tyro_g	Tyrosine (gm)	7,802	3.54 (0.04)	8,887	2.35 (0.03)
uap	Serum uric acid (mg/dL)	7,719	6.05 (0.02)	8,777	4.65 (0.03)
ubp	Urinary albumin (ug/mL)	7,888	28.91 (2.01)	8,885	25.09 (1.85)
urp	Urinary creatinine (mg/dL)	7,888	152.54 (1.84)	8,885	110.4 (1.61)
valin_g	Valine (gm)	7,802	5.05 (0.06)	8,887	3.39 (0.04)
vcella	Serum Varicella Antibody	7,688	13.07 (0.19)	8,701	12.53 (0.2)
veg_hei	Vegetables, HEI Score	7,802	6.08 (0.07)	8,887	5.99 (0.05)
vit_a	Serum viatmin A (ug/dL)	7,802	62.33 (0.39)	8,827	55.15 (0.37)
vit_a_g	Total vitamin A (IU)	7,802	6961.19 (153.34)	8,887	5885.49 (129.93)
vit_b12	Serum vitamin B12	3,718	467.13 (6.52)	4,826	497.68 (10.12)
vit_b6_g	Vitamin B6 (mg)	7,802	2.22 (0.02)	8,887	1.53 (0.02)
vit_c	Serum vitamin C (mg/dL)	7,420	0.67 (0.01)	8,408	0.82 (0.02)
vit_c_g	Vitamin C (mg)	7,802	100.12 (2.44)	8,887	83 (1.38)
vit_d_g	Vitamin D (mcg)	7,802	5.7 (0.14)	8,887	4.26 (0.12)
vit_e	Serum viatmin E (ug/dL)	7,802	1124.9 (11.68)	8,827	1174.48 (11.88)
vit_e_g	Total alpha-tocopherol equivalents (mg)	7,802	11.66 (0.22)	8,887	8.55 (0.2)
vitb12_g	Vitamin B12 (mcg)	7,802	6.96 (0.33)	8,887	4.43 (0.22)
vprot_g	Vegetable Protein (gm)	7,802	28.91 (0.28)	8,887	20.69 (0.24)
water_g	Water (gm)	7,802	2456.11 (27.96)	8,887	1759.21 (19.04)
wbc	White blood cell count	7,866	7.26 (0.04)	8,929	7.29 (0.05)
y_cig	Year smoked this amount (years)	2,405	12.89 (0.31)	1,899	12.12 (0.35)
zinc_g	Zinc (mg)	7,802	14.42 (0.18)	8,887	9.49 (0.14)

Table S2. Associations between metabolites, nutrients, and lifestyle factors and regular coffee consumption in the discovery set. All models were adjusted for age, race/ethnicity, education, and poverty-to-income ratio (PIR). Benjamini-Hochberg adjusted *P* values for false discovery rate (FDR) <5% are shown.

Variable	Description	No.	Coefficient	SE [1]	*P* value
cig_100	Smoked 100+ cigarettes in life (yes, no)	7,756	22.05422	1.981932	1.37E-05
cig_now	Current smoking (vs. never smoking)	5,772	32.33245	2.841555	1.37E-05
cig_age	Age first smoked cigarettes regularly (<30, 30-40, 40-50, >50)	7,626	9.141577	0.873465	1.78E-05
water_g	Water (gm)	7,332	14.82378	1.832383	0.000205
shs_home	Does anyone smoke at home? (yes, no)	7,755	21.63089	2.847887	0.000312
cop	Serum cotinine (ng/mL)	6,994	13.4838	1.905335	0.000526

smoke_hm	Number of person who smoke at home	7,755	9.90006	1.434324	0.000576
lead	Serum Lead Concentration (ug/dL)	7,371	9.226645	1.40966	0.000842
cafe_g	Caffeine (mg)	7,332	25.30226	3.954956	0.00093
shs_work	Can smell smoking in office? (yes, no)	4,346	13.723	2.291669	0.001551
vit_c	Serum vitamin C (mg/dL)	6,878	-6.32099	1.067234	0.001559
pipe_20	Smoked 20 pipes of tobacco in life (yes, no)	7,750	19.54035	3.35904	0.001684
fruc_g	Fructose (gm)	7,330	-5.83195	1.013175	0.00171
cig_form	Former smoking (vs. never smoking)	5,615	11.69876	2.083361	0.001851
smoke_ofc	Hours per day can smell smoking at work	4,346	6.99508	1.244223	0.001851
p_carb	Energy from carbohydrate (%kcal)	7,332	-6.59884	1.194247	0.002003
cigar_20	Smoked at least 20 cigars in life (yes, no)	7,755	20.05989	3.795369	0.002407
fru_hei	Fruits, HEI Score	7,332	-5.11056	0.969535	0.002407
magn_g	Magnesium (mg)	7,332	7.051394	1.344589	0.002407
pots_g	Potassium (mg)	7,332	9.21719	1.745673	0.002407
vit_e	Serum vitamin E (ug/dL)	7,152	-4.45207	0.840058	0.002407
cig_day	Number of cigarettes smoked per day	2,005	17.46126	3.396121	0.002722
gluc_g	Glucose (gm)	7,332	-5.18618	1.054213	0.003772
folate_r	RBCs folate (ng/mL)	7,183	-4.7202	0.965533	0.003808
folate_s	Serum folate (ng/mL)	7,267	-4.79126	1.007307	0.004576
tbp	Serum total bilirubin (mg/dL)	7,061	-4.23156	0.897036	0.004705
heiscore	Healthy Eating Index Score	7,332	-5.4416	1.276992	0.010027
b_crypto	Serum beta cryptoxanthin (ug/dL)	7,152	-4.72136	1.134059	0.011508
cadmium	Urinary cadmium (ng/mL)	7,363	3.996799	1.03486	0.018692
niac_g	Niacin (mg)	7,332	5.318667	1.379637	0.018692
aspirin	Took aspirin in last month (yes, no)	7,692	6.352288	1.750871	0.027368
shs	Passive smoking (yes, no)	3,631	7.679238	2.292631	0.044293
chol_g	Cholesterol (mg)	7,332	3.766012	1.168731	0.054368
fbp	Plasma fibrinogen (mg/dL)	4,011	3.604428	1.126619	0.055067
alc_mo	Alcohol consumption per month	7,735	4.054021	1.274561	0.055374
hsv2	Serum Herpes II Antibody	6,861	7.323997	2.346252	0.058708
leuc_g	Leucine (gm)	7,332	3.738315	1.198443	0.058708
p_tfat	Energy from total fat (%kcal)	7,332	3.57777	1.155603	0.059691
anprot_g	Animal Protein (gm)	7,332	3.465481	1.129238	0.060225
aspac_g	Aspartic Acid (gm)	7,332	3.991025	1.310543	0.060225
cvd	Cardiovascular disease (yes, no)	7,689	-13.9941	4.572232	0.060225
sscystat	Cystatin C (mg/L)	2,514	-4.64195	1.52828	0.060225
cig_dmax	Number of cigarettes per day when smoking most	685	17.80718	5.925407	0.060414
fat_hei	Fats, HEI Score	7,332	-3.8824	1.286141	0.060414
mfa161_g	MFA 16:1 (gm)	7,332	3.139286	1.048864	0.060414
sfa160_g	SFA 16:0 (gm)	7,332	3.535567	1.183759	0.060414
lysin_g	Lysine(gm)	7,332	3.422599	1.192231	0.068574
p_msfat	Energy from monosaturated fat (%kcal)	7,332	2.833782	0.986031	0.068574
p_sfat	Energy from saturated fat (%kcal)	7,332	3.463389	1.216279	0.068574
pfa183_g	Linolenic acid (PFA 18:3) (gm)	7,332	3.519936	1.240021	0.068574
prot_g	Protein (gm)	7,332	3.568866	1.231491	0.068574

sfa_hei	Saturated Fat, HEI Score	7,332	-3.11346	1.089309	0.068574
sfa180_g	SFA 18:0 (gm)	7,332	3.56122	1.230394	0.068574
totfat_g	Total fats (gm)	7,332	3.424138	1.209007	0.068574
p_alc	Energy from alcohol (%kcal)	7,332	2.709944	0.975336	0.07438
alan_g	Alanine (gm)	7,332	3.404358	1.27908	0.078014
alco_g	Alcohol (gm)	7,332	2.679958	0.978891	0.078014
b_carot	Serum beta carotene (ug/dL)	7,152	-2.76225	1.033956	0.078014
copp_g	Copper (mg)	7,332	3.073132	1.158411	0.078014
dm	Diabetes mellitus (yes, no)	7,739	-9.30913	3.471558	0.078014
glyc_g	Glycine (gm)	7,332	3.49689	1.322951	0.078014
meth_g	Methionine (gm)	7,332	3.113166	1.164926	0.078014
mfa181_g	Oleic acid (MFA 18:1) (gm)	7,332	3.128231	1.172756	0.078014
mufat_g	Total monounsaturated fatty acids (gm)	7,332	3.240092	1.196951	0.078014
satfat_g	Total saturated fatty acids (gm)	7,332	3.361994	1.236793	0.078014
thre_g	Threonine (gm)	7,332	3.185467	1.207523	0.078014
tryp_g	Tryptophan (gm)	7,332	3.185467	1.207523	0.078014
uap	Serum uric acid (mg/dL)	7,061	-3.53353	1.337354	0.078014
lipiddrug	Under lipid lowering treatment (yes, no)	3,448	-16.7937	6.449566	0.079259
shs_cop	Passive smoking by COP (yes, no)	3,267	6.656896	2.561875	0.079259
tyro_g	Tyrosine (gm)	7,332	3.010835	1.158677	0.079259
valin_g	Valine (gm)	7,332	3.108936	1.195175	0.079259
isoleu_g	Isoleucine (gm)	7,332	3.000838	1.159918	0.079823
argin_g	Arginine (gm)	7,332	3.27304	1.27728	0.082394
systole	Systolic blood pressure	7,510	-3.21536	1.270484	0.086175
histi_g	Histidine (gm)	7,332	3.190144	1.270418	0.088171
mfa201_g	MFA 20:1 (gm)	7,332	3.349364	1.341369	0.088171
phen_g	Phenylalanine (gm)	7,332	3.014093	1.205089	0.088171
glucose	Serum glucose (mg/dL)	7,061	-2.32947	0.937212	0.088901
p_prot	Energy from protein (%kcal)	7,332	2.424793	0.979806	0.089538
mea_hei	Meats, HEI Score	7,332	1.904435	0.78421	0.095075
serin_g	Serine (gm)	7,332	2.889362	1.187251	0.095075
dwp	Platelet distribution width (%)	7,163	-2.55459	1.067186	0.097674
mfa141_g	MFA 14:1 (gm)	7,332	4.037891	1.679978	0.097674
urp	Urinary creatinine (mg/dL)	7,306	-3.12102	1.307185	0.097674
vitb12_g	Vitamin B12 (mcg)	7,332	3.031258	1.268599	0.097674
lmp	Lymphocyte number	7,201	2.850787	1.199408	0.098462
cep	Serum creatinine (mg/dL)	7,061	-2.84652	1.208305	0.10114
ca_norm	Serum normalized calcium (mmol/L)	6,142	-2.2118	0.951298	0.105749
insulin	Serum Insulin	6,793	-2.72061	1.185312	0.107968
lut_zea	Serum lutein zeaxanthin (ug/dL)	7,152	-2.10803	0.914432	0.107968
monop	Mononuclear percent	7,097	-1.84103	0.801362	0.107968
glutac_g	Glutamic Acid (gm)	7,332	2.866298	1.254481	0.10884
rubella	Serum Rubella Antibody IU	6,972	2.024093	0.89035	0.109939
sfiber_g	Water soluble dietary fiber (gm)	7,332	3.4116	1.506973	0.110662
bup	Serum blood urea nitrogen (mg/dL)	7,061	-2.63254	1.174767	0.112962

ca_tot	Serum total calcium (mmol/L)	6,956	-1.83277	0.817812	0.112962
igp_bp3	Insulin like growth factor binding protein-3 (ng/ml)	2,423	-2.48992	1.114936	0.113363
ssb2m	Beta-2 microglobulin (mg/L)	2,543	-3.30535	1.498743	0.117975
tip	Serum TIBC (ug/dL)	7,264	-2.1711	0.987064	0.118029
ash_g	Ash (gm)	7,332	3.089435	1.41669	0.120869
iodine	Urinary iodine (ug/dL)	7,227	-0.48859	0.227759	0.126297
tpp	Serum total protein (g/dL)	7,061	-2.18552	1.018592	0.126297
pct_mo	Amount of paracetamol takes past month	2,646	6.925394	3.24889	0.128149
cyst_g	Cystine (gm)	7,332	2.558964	1.264058	0.153471
pfa204_g	PFA 20:4 (gm)	7,332	2.623759	1.311013	0.158316
pufat_g	Total polyunsaturated fatty acids (gm)	7,332	2.083304	1.052996	0.163248
lep	Serum leptin	2,523	-3.375	1.757397	0.178938
atpsi	ALT_Alanine aminotransferase (U/L)	7,061	-1.77929	0.943399	0.188179
a_carot	Serum alpha carotene (ug/dL)	7,152	-2.36846	1.27685	0.193293
aspar_g	Aspartame (mg)	7,332	-1.42238	0.766905	0.193293
sfa140_g	SFA 14:0 (gm)	7,332	2.441513	1.311444	0.193293
ssbtp	Beta-trace protein (mg/L)	2,542	-2.63227	1.470185	0.211959
wbc	White blood cell count	7,203	2.379152	1.326126	0.211959
pfa225_g	PFA 22:5 (gm)	7,332	1.499292	0.853685	0.220729
tgp	Serum triglycerides (mg/dL)	7,201	-1.61009	0.915798	0.220729
malt_g	Maltose (gm)	7,332	-1.92595	1.107554	0.223432
sele_g	Selenium (mcg)	7,332	1.978429	1.137663	0.223432
pfa226_g	PFA 22:6 (gm)	7,332	1.069731	0.619652	0.226295
pfa182_g	Linoleic acid (PFA 18:2) (gm)	7,332	1.798718	1.048164	0.228317
kcal	Calories (kcal)	7,332	2.024934	1.191158	0.232619
proli_g	Proline (gm)	7,332	2.107967	1.257095	0.239789
cancer	History of cancer (yes, no)	7,754	-5.15989	3.097108	0.240208
sum_ret	Serum sum retinyl esters (ug/dL)	7,152	-0.67844	0.406922	0.240208
dmdrug	Under diabetes treatment (yes, no)	3,456	-6.08598	3.676869	0.242609
mvpsi	MCV	7,202	1.914726	1.172787	0.249847
zinc_g	Zinc (mg)	7,332	1.591718	0.979729	0.251164
carb_g	Total carbohydrates (gm)	7,332	-2.03195	1.259933	0.254134
fcig_day	Number of cigarettes per day before quitting	1,871	2.78857	1.756287	0.26271
appsi	ALP_Serum alkaline phosphatase (U/L)	7,059	-1.692	1.088991	0.273669
mcpsi	MCH	7,202	2.113338	1.360597	0.273669
sk	Serum potassium (mmol/L)	7,061	1.564106	1.010744	0.274148
aspsi	AST_Aspartate aminotransferase (U/L)	7,061	-1.60172	1.045615	0.279059
cho_hei	Cholesterol, HEI Score	7,332	-1.78709	1.183929	0.283049
ht	Hypertension (yes, no)	7,738	-3.79939	2.517497	0.283049
vit_c_g	Vitamin C (mg)	7,332	-2.02798	1.34283	0.283049
apoa	Serum apolipoprotein A1 (mg/dL)	7,178	-1.5719	1.049712	0.286324
scl	Serum chloride (mmol/L)	7,061	1.408836	0.94437	0.286827
ha_ab	Serum Hepatitis A Antibody	6,980	4.858773	3.448609	0.324747
nsaid	Took NSAID in last month (yes, no)	7,696	3.279333	2.337869	0.325578
rwp	Red cell distribution width (%)	7,203	1.647733	1.194206	0.335048

ldpsi	LDH_Serum lactate dehydrogenase (U/L)	7,061	-2.02785	1.516221	0.355212
ribo_g	Riboflavin (mg)	7,332	1.560132	1.173372	0.356994
d_toco_g	Delta-tocopherol (mg)	7,332	0.788175	0.612512	0.373267
pct	Took paracetamol in last month (yes, no)	7,698	-3.19331	2.475058	0.373267
phos_g	Phosphorous (mg)	7,332	1.624288	1.261286	0.373267
retino_g	Retinol (mcg)	7,332	1.439239	1.134844	0.381283
sfa100_g	SFA 10:0 (gm)	7,332	1.68146	1.332162	0.382159
p_psfat	Energy from polysaturated fat (%kcal)	7,332	1.266515	1.00778	0.382728
lact_g	Lactose (gm)	7,332	1.377767	1.11215	0.390519
fiber_g	Dietary fiber (gm)	7,332	1.500755	1.220775	0.392359
grp	Granulocyte number	7,097	1.825326	1.487871	0.392359
rubella_cat	Serum Rubella Categorical	6,803	5.317692	4.397608	0.397509
sfa40_g	SFA 4:0 (gm)	7,332	1.678555	1.384406	0.397509
phyt_g	Phytic Acid (mg)	7,332	1.19344	0.991941	0.398518
iron_g	Iron (mg)	7,332	1.299873	1.085113	0.399029
gra_hei	Grains, HEI Score	7,332	-1.34	1.130514	0.400166
hba1c	Glycated Hemoglobin	7,319	1.309115	1.106562	0.400166
igp_i	Insulin like growth factor-I (ng/ml)	2,424	-2.27594	1.913836	0.400166
meet_yr	Meetings with friends,<br>relatives or neighbors at least once a week (yes, no)	7,755	5.544103	4.808083	0.418519
diastole	Diastolic blood pressure	7,510	1.285651	1.159323	0.440049
b_toco_g	Beta-tocopherol (mg)	7,332	-0.91628	0.848161	0.452778
sacch_g	Saccharin (mg)	7,332	0.743109	0.687571	0.452778
sfa60_g	SFA 6:0 (gm)	7,332	1.497003	1.410351	0.462192
frp	Serum ferritin (ng/mL)	7,266	-1.25268	1.202348	0.469354
sfa120_g	SFA 12:0 (gm)	7,332	1.016988	0.97333	0.469354
hdl	HDL cholesterol (mg/dL)	7,171	1.130724	1.096922	0.471376
sfa220_g	SFA 22:0 (gm)	7,332	-0.73944	0.720738	0.471376
sna	Serum sodium (mmol/L)	7,061	-0.96006	0.935546	0.471376
sfa80_g	SFA 8:0 (gm)	7,332	0.907422	0.895865	0.477167
pfa184_g	PFA 18:4 (gm)	7,332	0.383262	0.385852	0.487388
aspi_mo	Amount of aspirin taken past month	2,790	-1.28914	1.314117	0.492795
fola_g	Folic acid (mcg)	7,332	1.427552	1.476608	0.49954
phosp	Serum phosphorus (mg/dL)	7,061	1.290779	1.341721	0.499883
vit_a	Serum viatmin A (ug/dL)	7,152	-0.95559	1.001091	0.502095
nsa_mo	Amount of NSAID takes past month	1,302	1.859943	2.008103	0.518656
hsv1	Serum Herpes I Antibody	6,893	2.239795	2.447429	0.5234
vit_b6_g	Vitamin B6 (mg)	7,332	1.059024	1.168438	0.526592
g_toco_g	Gamma-tocopherol (mg)	7,332	0.664798	0.738379	0.527842
mhp	MCHC	7,202	0.888027	1.008966	0.539096
sod_hei	Sodium, HEI Score	7,332	-1.01141	1.185145	0.555171
lymp	Lymphocyte percent	7,201	1.039257	1.238527	0.562439
bcaro_g	Beta-carotene (mcg)	7,332	-0.69365	0.869023	0.568974
crp	Serum C-reactive protein (mg/dL)	7,135	-1.06845	1.319769	0.568974
epp	Erythrocyte protoporphyrin (ug/dL)	7,334	1.51324	1.84187	0.568974

hgp	Hemoglobin (g/dl)	7,203	1.293512	1.616559	0.568974
pantho_g	Pantothenic acid (mg)	7,332	0.885206	1.111444	0.568974
tcp	Serum cholesterol (mg/dL)	7,235	0.954198	1.178322	0.568974
tetanus	Serum Tetanus Antibody	7,099	0.682565	0.8526	0.568974
vit_d_g	Vitamin D (mcg)	7,332	0.748796	0.916499	0.568974
lbdvidms	Vitamin D (nmol/L)	7,082	-0.80181	1.043837	0.586536
ldl	LDL cholesterol (mg/dL)	3,050	1.752256	2.372081	0.605214
hc_ab	Serum Hepatitis C Antibody	6,949	5.766704	7.879397	0.607165
pect_g	Pectin (gm)	7,331	-0.8098	1.161687	0.630217
mfa221_g	MFA 22:1 (gm)	7,332	0.687711	0.998003	0.633074
tmp	Serum antimicrosomal antibody (U/mL)	6,851	-0.35565	0.524445	0.638183
calc_g	Calcium (mg)	7,332	0.718904	1.100214	0.64735
hppcag	H Pylori CagA Seropositivity	6,621	1.234519	1.868741	0.64735
sfe	Serum iron (ug/dL)	7,281	-0.78715	1.204261	0.64735
ifiber_g	Water insoluble dietary fiber (gm)	7,332	0.676952	1.049771	0.650242
pfa205_g	PFA 20:5 (gm)	7,332	0.593581	0.925772	0.650242
pltp	Platelet Count	7,200	0.719901	1.140411	0.654594
hctp	Hematocrit (%)	7,202	0.949288	1.66372	0.69175
l1p	Serum latex antibody (IU/mL)	4,786	-0.58826	1.024257	0.69175
pvpsi	Mean platelet volume	7,204	0.38511	0.672068	0.69175
sucr_g	Sucrose (gm)	7,332	-0.7017	1.260542	0.699306
sodi_g	Sodium (mg)	7,332	0.658901	1.218938	0.705206
thp	Serum TSH (uU/mL)	6,851	-0.88479	1.634696	0.705206
y_cig	Year smoked this amount (years)	2,043	1.421653	2.661796	0.706901
mop	Mononuclear number	7,097	-0.46392	0.878304	0.708155
hbs_ab	Serum Hepatitis B Surface Antibody	438	4.183926	8.254086	0.722503
chewsnuf	Chewing tobacco or snuff (yes, no)	7,756	-1.82498	4.284468	0.779494
rcp	RBCs count	7,202	-0.72106	1.68042	0.779494
sfa170_g	SFA 17:0 (gm)	7,332	0.272001	0.645392	0.779509
gala_g	Galactose (gm)	7,332	0.397486	1.008607	0.798158
t4p	Serum thyroxine (ug/mL)	6,593	0.486361	1.298072	0.810357
hbc_ab	Serum Hepatitis B Core Antibody	6,985	1.016014	2.754642	0.811407
oxal_g	Oxalic Acid (mg)	7,332	-0.3779	1.114194	0.829035
rfp	Serum rheumatoid factor antibody	2,255	-0.22863	0.676007	0.829035
toxo	Serum Toxoplasmosis Antibody	6,470	0.37405	1.134654	0.832265
cfiber_g	Crude Fiber (gm)	7,332	-0.31613	0.988386	0.836533
htdrug	Under hypertension treatment (yes, no)	3,448	0.959047	3.047846	0.836996
granp	Granulocyte percent	7,097	-0.39524	1.282356	0.838499
tap	Serum antithyroglobulin antibody (U/mL)	6,851	-0.09361	0.321885	0.848928
ubp	Urinary albumin (ug/mL)	7,306	-0.31179	1.158878	0.862905
selenium	Serum selenium (ng/mL)	6,990	-0.25353	1.006773	0.873148
shco3	Serum bicarbonate (mmol/L)	7,061	-0.25835	1.055984	0.875152
veg_hei	Vegetables, HEI Score	7,332	-0.21109	0.914304	0.88254
vcella	Serum Varicella Antibody	6,972	0.262244	1.16815	0.883871
dai_hei	Dairy, HEI Score	7,332	0.269581	1.275592	0.890703

age_stop	Age last smoked cigarettes regularly	1,946	0.760246	3.926102	0.894748
amp	Serum albumin (g/dL)	7,061	0.233544	1.279607	0.894748
bmi	BMI	7,724	-0.21771	1.18686	0.894748
star_g	Starch (gm)	7,332	-0.25838	1.396194	0.894748
vprot_g	Vegetable Protein (gm)	7,332	-0.23517	1.282092	0.894748
a_toco_g	Alpha-tocopherol (mg)	7,332	-0.12134	0.772049	0.911197
pxp	Serum transferrin saturation (%)	7,246	-0.14209	1.054885	0.925301
apob	Serum apolipoprotein B (mg/dL)	7,200	0.141092	1.18608	0.930055
hpylori	Serum Helicobacter Pylori Antibody	6,919	0.310389	2.541495	0.930055
phone_wk	Daily phone calls with friends, relatives or neighbors (yes, no)	6,651	-0.31944	2.851174	0.93183
thia_g	Thiamin (mg)	7,332	0.109818	1.577058	0.957354
vit_a_g	Total vitamin A (IU)	7,332	0.085516	1.176575	0.957354
vit_e_g	Total alpha-tocopherol equivalents (mg)	7,332	0.049704	0.780655	0.958103
lycopene	Serum lycopene (ug/dL)	7,152	-0.05751	1.152714	0.961032
sfa200_g	SFA 20:0 (gm)	7,332	-0.05192	0.950532	0.961032

[1] Standard Error

Table S3. Validated Metabolites, Nutrients and Lifestyle Factors of Regular Coffee Consumption and Their Association With Coffee Consumption (continuous) Stratified by Sex [a]

	men			women		
	Coefficient	SE[1]	Pvalue	Coefficient	SE	Pvalue
Water (gm) [2]	16.48	3.56	4.72E-04	11.04	2.56	8.43E-04
Caffeine (mg) [2]	23.31	2.01	3.17E-08	20.05	4.52	6.73E-04
Smoked 100+ cigarettes in life (yes, no)	21.46	2.71	2.48E-06	22.75	4.4	1.81E-04
Age first smoked cigarettes regularly (<30, 30-40, 40-50, >50)	9.44	1.18	2.31E-06	10.25	2.29	6.27E-04
Number of cigarettes smoked per day	14.08	6.25	4.22E-02	--		
Former smoking (vs.- never smoking)	9.1	2.57	3.63E-03	12.85	4.05	7.29E-03
Current smoking (vs. never smoking)	33.67	3.65	4.55E-07	36.08	6.82	1.45E-04
Smoked at least 20 cigars in life (yes, no)	--			13.35	5.65	3.45E-02
Smoked 20 pipes of tobacco in life (yes, no)	69.64	24.21	1.30E-02	18.75	7.31	2.35E-02
Serum cotinine (ng/mL)	12.83	1.46	7.92E-07	11.93	2.87	1.14E-03
Does anyone smoke at home? (yes, no)	23.2	2.76	1.29E-06	19.48	3.12	2.96E-05
Can smell smoking in office? (yes, no)	13.7	5.31	2.29E-02	17.6	5.32	5.65E-03
Number of persons who smoke at home	12.68	1.4	5.82E-07	10.03	2.26	6.79E-04
Hours per day can smell smoking at work	7.99	2.78	1.31E-02	8.5	2.84	1.04E-02
Took aspirin in last month (yes, no)	--			--		
Serum Lead Concentration (ug/dL)	9.39	1.58	4.76E-05	8.21	2.42	4.84E-03
Urinary cadmium (ng/mL)	--			12.21	2.41	2.17E-04
Serum total bilirubin (mg/dL)	-4.65	1.08	8.59E-04	-4.85	1.98	2.93E-02
Serum beta cryptoxanthin (ug/dL)	-4.3	1.01	9.42E-04	-4.9	1.47	5.37E-03
RBCs folate (ng/mL)	-4.32	0.67	2.16E-05	-8.61	1.25	1.11E-05
Serum folate (ng/mL)	--			--		
Serum vitamin C (mg/dL)	-2.82	1.19	3.41E-02	-5.92	1.92	8.68E-03
Serum vitamin E (ug/dL)	-3.05	0.76	1.46E-03	--		
Fruits, Healthy Eating Index Score[2]	-3.84	1.02	2.35E-03	-4.16	1.73	3.17E-02
Healthy Eating Index Score[2]	-5.7	1.13	2.28E-04	-4.24	1.82	3.62E-02
Energy from carbohydrate (%kcal) [2]	--			-6.97	1.76	1.62E-03
Fructose (gm) [2]	-5.78	0.91	2.51E-05	-4.89	1.35	3.04E-03
Glucose (gm) [2]	-4.79	0.86	8.98E-05	-5.21	1.59	6.05E-03
Magnesium (mg) [2]	5.57	2.33	3.26E-02	3.68	1.67	4.62E-02
Potassium (mg) [2]	7.4	2.68	1.62E-02	4.39	1.52	1.28E-02

[1] Abbreviations: gm, gram; mg, milligram; RBCs, red blood cell; SE, standard error;
[2] Assessed from 24-hour dietary recall interviews
